# Dentistry and Drug Adverse Events: Between Responsibilities and Regulations

**DOI:** 10.3390/toxics10110671

**Published:** 2022-11-07

**Authors:** Tommaso D’Anna, Antonina Argo, Stefania Zerbo, Diego D’Urso, Maria Sofia Rini

**Affiliations:** 1 Policlinic Hospital, University of Palermo, Via del Vespro 129, 90127 Palermo, Italy; 2 Department of Health Promotion, Mother and Child Care, Internal Medicine and Medical Specialties, Section of Legal Medicine, University of Palermo, 90127 Palermo, Italy; 3 Dentistry Faculty, University of Bologna, Via San Vitale 59, 40125 Bologna, Italy

**Keywords:** dentistry, drugs, appropriateness, toxicity, adverse events, errors, complications, medical–legal assessment

## Abstract

The purpose of this paper is to renew interest and attention to the medical history, prescription, and/or use of drugs during dental practice. The work analyzes the issue of the use of drugs in dentistry from both a clinical and a medical–legal point of view. The laws governing the matter were also taken into consideration, relating them to the roles of prescriber and user that the dentist can acquire. Analysis of various aspects of this matter demonstrates that it is necessary for dentists to know the drugs and medical substances, their characteristics and properties, related effects, and interactions in order to use them appropriately and adequately. Knowledge of interferences, reactions, and adverse events or complications helps to reduce errors and protect patients’ health. Furthermore, knowledge of the national and international reference standards relating to the use of drugs leads to a reduction in medico–legal questions. In conclusion, knowledge and adequate and appropriate use of drugs reduces the possibility of accidents, adverse events, medico–legal consequences, and disputes with patients. Documented and traceable choices allow the analysis and valuation of professional conduct. Authors perceived the topic of informed consent of patients as relevant to the adequate procedure of prescribing drugs related to dentistry practice; therefore, representative conditions of patients at risk should be illustrated in practice. Legal duties related to physician drug prescription and use must be considered and carefully checked.

## 1. Introduction

The term drug is derived from the Greek word φαρμακον, *pharmakon*, which has the dual meaning of medicine and poison. The benefits associated with the administration of medicinal substances or considered as such, have indeed been known since ancient times; likewise, it is well known the possibility that they may be ineffective or produce harmful or undesirable effects [1,2,3].

Dentists sometimes need to prescribe drugs that are not of direct interest to their branch or are even off-label—e.g., in the case of metronidazole, a gynecological drug that finds indications in this way during periodontal disease therapy.

In Italy, Law No. 409/1985, instituting the health profession of dentists, established that graduates in dentistry can prescribe “all medications necessary for the exercise of the profession”. While antibiotics, analgesics, anti-inflammatories, and local anesthetics are commonly used, on the other hand, the prescription or administration of drugs or medicinal substances with non-dental indications for use may appear less logical. Sometimes, however, these drugs are necessary—e.g., in the case of magnesium and muscle relaxants (clinical approach) in association with physiotherapy and muscle stretching in some gnathological activities, or of benzodiazepines in the pre- and/or post-surgery case in patients which are particularly agitated, anxious, having specific disabilities, or resorting to emergency medications.

There are categories of prescription drugs which dentists are authorized to prescribe, as in the case of atropine sulphate, the prescription of which is permitted pursuant to determination on 29 May 2012 by the Italian Medicines Agency (AIFA), the Ministry of Health, and National Federation of Orders of Physicians/Surgeons and Dentists.

Knowledge of drugs and medicaments used in health care must be deep and extensive, about trade names, active ingredients, excipients, carriers, indications for use, precautions, interactions with other drugs or other substances or foods, unwanted side effects, and anything else useful to safeguard the patient’s health. The precautionary procedures and the evaluation of the indications for use must be correct, appropriate, validated, and documented. The traceability and adequacy of choices, precautionary procedures, and the behavior of the caregiver represent elements of evaluation of clinical conduct. Failure or incorrect use of the product in the absence of correct and validated precautionary and evaluation procedures can demonstrate negligent conduct, even more so if causing damage.

Bromelain is an example of a drug with anti-edema and mild anti-inflammatory power, which can be used in the dental field. It is not indicated in hemophilic patients, with hemorrhagic diathesis, severe nephropathies, peptide ulcer, Lapp lactase deficiency or with glucose–galactose malabsorption, vegetarians, or patients with galactose intolerance. Furthermore, patients on anticoagulants or antithrombotic or antiplatelet drugs should not take bromelain to avoid an enhancement of this effect. Furthermore, bromelain can interfere with some antibiotics, including amoxicillin and tetracyclines. Allergic reactions can be triggered in patients with allergies to latex, pineapple, wheat, carrot, fennel, celery, papain, cypress, and grass pollen [4].

What is represented above identifies how dentists can certainly not limit themselves to knowing only the drugs exclusive for dental use, and the necessity for their knowledge to extend—albeit within the limits of their competence—to all drugs and medicaments, to avoid negative or unfortunate interferences with substances taken by the patient, or conditions not strictly of specific interest.

## 2. Patient Informed Consent

Administration of drugs and medicinal substances presupposes the informed expression of the patient’s adherence to the proposed treatment. Information about the foreseeable immediate and late consequences of the treatment to be subjected, with the known possibility of aggravation or non-alteration of the *quo ante* conditions, is necessary to consciously decide whether to adhere to it or not.

The collection of valid informed consent—also in relation to the administration, prescription, and/or use of drugs or medicinal substances—is a legal obligation. Problems relating to the issue of consent arise in all cases in which no positive changes are associated with the therapy or aesthetic dental procedure, or in which unwanted events or specific damage related to the substance used arise [5,6,7,8].

The patient must be informed about the possibility that even their inadequate conduct that does not comply with the instructions of the prescription of the healthcare professional may interfere with the pharmacological action. In the dental field, a typical example can be represented by the appearance and aggravation of hyperplastic gingivitis associated with the administration of diphenylhydantoin, favored by unsuitable home hygienic maintenance [9].

There are also cases in which the pharmacological or medicinal substance is used for serious and disabling pathologies, where, moreover, causal cooperation of clinical skills and technical responsibilities can be found that can be correlated to different medical specialties. An example is that of the appearance of osteonecrosis of the jaw bones, fractures of the femur, or gastric carcinoma [10,11,12] in patients taking bisphosphonates which is a composite category of drugs that is different in concentration and indications of use, widely used to counteract the loss of bone mineral density both in the case of prevention and reduction of damage from osteoporosis, and in some serious neoplastic pathologies. When considering bisphosphonates, practical, clinical, managerial, and medico–legal problems arise, not only in relation to their prescription and administration, but also to questions of appropriateness of therapeutic choice, of prophylactic and preparatory interventions for drug administration, of correct identification and early management of any osteonecrosis complication [13]. Any intervention by the dentist, if not preliminary and preventive remediation, must consider a multiplicity of factors related to the intervention to be implemented, the type and timing of administration of the therapy, the prophylactic protocols for the bloody intervention, and to the obligations of surveillance and early identification of osteonecrosis findings [14]. The patient must be made aware of the risk, and participate in therapeutic choices and prophylactic behaviors. In the event of a lack of information, of non-validated prophylactic procedures, of failure to identify and recognize the lesions early, or of incorrect management of the complication, the damage and/or worsening of the clinical picture would constitute the extremes of gross negligence due to imprudence, lack of diligence, and sanitization procedures.

In case of an accident or a negative event, particular value for the purposes of any insurance coverage assumes the legitimacy of the use of substances, also supported by valid informed consent. Obviously, actions in violation of the law or the illegitimate use of medicinal substances, exclude the possibility of insurance coverage.

One of the classic examples relates to the use of an endodontic canal sealer used in dentistry, medicated with endomethasone. This drug, now no longer in use due to its harmful effects, is still marketed and is legitimate to use, but this use requires diligence, expertise and technical skill, the absence of greater safety alternatives, and specific and in-depth information on its potential harmful effects. The existence of products and methods of greater reliability and safety renders the use of the product negligent. Cases of irreversible injuries to the inferior alveolar nerve (NAI) from the introduction of endomethasone into the alveolar canal with recognition of negligent conduct by the dentist, in a causal link with the neurological damage produced, are documented in the literature [15,16].

A careful evaluation deserves the existence of physiological conditions, such as pregnancy, pediatric or geriatric age in the context of which the reference evidence is scarce or, in any case, precautionary. Careful evaluation deserves pathological states associated or not with other pharmacological assumptions. The professional’s conduct must refer to the knowledge or availability of scientific–clinical evidence and AIFA indications related to the specific case. In such circumstances, the omission of the relevant annotation in the clinical diary of the therapeutic decisions taken, the related reasons and consequent outcomes would be particularly serious.

The national annual report on the use of drugs [17], by the Ministry of Health and Social Policies, provides useful knowledge and tools in order to improve the appropriateness of use of drugs, also identifying areas of variability through specific indicators. The latter often has a strictly economic and political value to contain public spending but promote updates on prescriptive appropriateness. The healthcare professional compares this evidence with the needs of the specific case and finds in the system indicators, interesting insights, and comparisons with the experiences of other professionals.

## 3. Drug Prescription

The prescription of a drug is a legally relevant medical act. It is a “complex practice”, a source of responsibility, which underlies a delicate clinical anamnestic/diagnostic process, but also administrative, economic (e.g., drugs charged to the NHS) and documentary type.

Through the prescription, the healthcare professional performs a recognition activity of the client’s subjective right to supply medicines, making this right operational with the issue of a document, which assumes the complex nature of a certificate [18]. The act of prescribing presupposes accurate diagnostic assessments, an act that must meet specific canons and formal requirements [18,19,20]. The therapeutic prescription must be completed and signed by the physician/surgeon/dentist by hand, digitally, or in any case by means of an indelible, non-computer-modifiable, and clearly legible method, and without corrections. Any corrections must be countersigned. The identifying details and titles of the prescriber, the patient’s name and surname, the name of the active ingredient or drug must be indicated, with the indication “not replaceable” if deemed necessary for valid and justified conditions, place and date of compilation, and a signature of the prescriber.

It is not mandatory, but it is advisable and synonymous with diligence, the indication of the pharmaceutical form, formulation, dosage, and dosage of the preparation [21]. Reference to pediatric or adult formulations is highly advisable to avoid situations in which such omissions may be related to adverse events.

The document may also contain dietary advice and indications of treatment with relative dosage and methods of use [1]. Technically, the prescription has the function of minimizing the risks associated with the use, administration, and intake of drugs to protect health and, secondly, for drugs provided by the public health system to contain public spending [12,16]. In the view of the medical iatrogenic, effort must be provided to ensure safety of care, and that access to the Pharmacovigilance system by physicians and health care professionals represents a significant measure of preventable adverse events [1,2].

## 4. Pharmacovigilance

In Italy, the AIFA (Italian Drugs Agency) provides that medicines, before being placed on the market, are authorized by the EMA (European Medicines Agency). After this step, the AIFA authorizes its marketing and use in Italy, providing that the substance placed on the market is accompanied by a specific technical data sheet with indication of the instructions relating to the product: composition, uses, interactions, contraindications, dosage, side effects known/unwanted, and precautions. The same drug can be included in the components of several medicines.

Since 1965, the criteria and procedures for authorizing the marketing of medicinal products have been the subject of extensive harmonization work at the European level. From 1993 until today, community authorizations have progressively become the point of reference.

Pharmacovigilance activities are aimed at promoting an appropriate and safe use of drugs to reduce possible risk factors, prevent, and minimize them. Whenever an alarm signal emerges regarding a drug, medicinal substance, or a class of drugs, it is necessary to identify and quantify the possible risk factors to effectively prevent unwanted effects. These interventions may lead to a change in the characteristics of the product or its use (restriction of indications, addition of contraindications and/or warnings), prescription, or dispensing, until the suspension or revocation by the marketing authority.

An essential element of this process is the widespread dissemination to all health professionals of the evidence found with, at times, the involvement of the citizen as well. The General Management of the Evaluation of Medicines and Pharmacovigilance of the Ministry of Health promotes data collection and dissemination actions through a series of paper and electronic means, among which we remember the DDL (Dear Doctor Letter) of the 1990s-2000s, communications addressed to physicians and sometimes, pharmacists, with content agreed or approved by the national or European authority in the context of the Pharmacovigilance Working Party or the meetings of the CPMP (Committee for Proprietary Medicinal Products) regarding substantial changes in the methods of use of a certain drug, of national or international discovery of new undesirable effects or abuses of the product.

In 1960 the FDA (Food and Drug Administration) in the United States promoted the Adverse Drug Reactions (ADR) registry and launched the first in-hospital pharmacovigilance programs (John Hopkins Hospital and Boston Hospital) [22]. In Italy, the pharmacovigilance system was born with Legislative Decree No. 44/1997. The National Pharmacovigilance System has been established which, through the Ministry of Health, relates to the EMA. In 1993 the Drug Safety Committee decided to monitor the safety of the substances placed on the market and to develop strategies to minimize the risks and increase the benefits associated with their use. Communications began on a large scale through AIFA, Local Health Unit Agencies, scientific societies, and a network of specific journals and sites. Monitoring, meta-analysis, and dissemination of data increase the basic knowledge of healthcare professionals and improve therapeutic standards, thus reducing the chances of ADR [4].

The culture of awareness of drug-related risk is consolidated. Thus, the concept of “acceptable risk” was born through knowledge, long-term evidence and continuous updating of available databases including those of AIFA and EMA.

The pharmacovigilance service periodically releases the most recent evidence [23]. This service is based on a very rigid process of pre-marketing verifications and validations [3] and in the post-marketing period to evaluate adverse or otherwise unwanted events reported by healthcare professionals [23].

### 4.1. Adverse Drug Reactions

ADR is defined as any harmful, unintended reaction to a drug administered for any purpose (preventive, diagnostic, or therapeutic) [24]. Drugs commonly used in dentistry practice are related to side effects that must be known and considered by health care professionals (Table 1). We report in Table 1 the pathological conditions, drugs, and possible adverse events related to drugs commonly prescribed in dentistry practice.

Since 2012, this concept also includes harmful and unwanted effects resulting from the administration or use of a medicine in accordance with the indications of the technical data sheet and the marketing authorization in European countries (EMA) or in Italy (AIFA), as well as the associated exposure for occupational uses [25].

There are known so-called “abnormal” type A reactions (predictable, dose-dependent), and “bizarre” type B reactions (unpredictable, non-dose-dependent). The latter usually occurs in predisposed subjects, hence the relevance of the anamnestic data and the results of pharmacogenetic and pharmacogenomic studies [26,27,28,29,30].

Constant specific updates published by AIFA and related documentation and all issues and disputes relating to drugs are available on the internet on the AIFA website.

AIFA notes are an important and validated practical and regulatory tool, aimed at defining the areas of responsibility of some medicines and define, among other things, the appropriateness in choosing a specific drug based on the data of the trial, on the evidence, and international literature. Furthermore, they report updates on the risks and benefits of drugs on the market based on the ADR reports of individual active ingredients or drugs received and the most common interactions between foods (foods or drinks) and drugs.

EudraVigilance is the European database for the management and analysis of reports of suspected adverse reactions to medicines that are authorized, or that are being studied through clinical trials, in the EEA (European Economic Area). The system has been operational since December 2001. Table 1 shows some examples of drugs commonly used in dentistry for which adverse reactions have been reported in the EudraVigilance database.

It is worthy to consider, regarding the routinely dentistry practice, the case of adverse drug reactions caused by a local vasoconstrictor/sympathomimetic, as a potential life threating side-effect [31,32,33,34]. Examples are not exhaustive, though are supported by evidence of number of adverse events, as indicated in Table 2. Worthy of consideration are the number of reports on Denosumab, an Anti-bone resorption monoclonal antibody (Table 2).

The reports to AIFA (Legislative Decree No. 219/2006) are made through a specific form, established in 2003 and updated in 2004, with the Ministry of Health Decree (Dir. EU No. 2010/84/EC) and downloadable from the website of the Agency (https://aifa.gov.it accessed on 25 October 2022).

Art. 36 co. 14 of Law No. 449/1997 provides for a specific drug information program: Pharmacovigilance and Health Education, to improve the appropriateness and efficiency of prescriptions and the use of drugs, while at the same time reducing public spending through specific, coordinated, and integrated projects.

The Ministry of Health, through the Offices of Maritime, Air and Border Health, supervises and verifies the import of drugs from abroad. In Italy it is possible to import and market and use only drugs approved and authorized by AIFA. However, according to the Legislative Decree No. 44/1997, a rule that regulates the methods of importing medicinal specialties registered abroad and limited to the possible authorized uses for them, and the possibility of requesting to import drugs authorized abroad, even outside the European Community. The problem has recently arisen of the high number of requests relating to the drug Parvulan, authorized by the Brazilian Regulatory Authority (ANVISA—*Agência Nacional de Vigilância Sanitária*, National Health Surveillance Agency), the subject of the request for the treatment of patients with clinical manifestations of Herpes Zooster. Among the therapeutic indications of Parvulan and authorized in Brazil are the stimulations of natural immunity, to act as an adjunct in the treatment of dermatological, systemic, and local infections of viral, bacterial origin (including Erysipelas from *Streptococcus pyogenes* and acne), fungal and protozoal, having a regressive effect on solid neoplasms [35]. The increase in the number of requests led to a ministerial assessment, which verified a different use of the medicine (off-label) from that indicated on the import request and outside the constraints of the Ministry of Health Legislative Decree No. 44/1997. The evidence relating to the use of Parvulan as an alternative to drugs authorized in the COVID-19 protection and prevention campaign was not deemed sufficient by the Scientific Technical Commission (CTS) of AIFA to authorize its use, even of an experimental type (Italian Medicines Agency: https://www.aifa.gov.it/en/riassunto-caratteristiche-e-foglio-illustrativo accessed on 25 October 2022).

### 4.2. Pharmacogenetics and Pharmacogenomics

The same dosage of a drug, on different patients, but with the same diagnosis and type of prescription, produces in individuals different responses (good, little, or none) or even side effects or toxic [35,36]. Most deaths caused by individual drug response variability are largely genetic and ADRs are the fourth leading cause of death after cardiovascular disease [37,38,39,40].

There are several factors that influence the response to drugs: correctness of diagnosis, patient compliance, age, sex, body weight, liver and/or kidney function, interaction with other drugs or substances, other diseases, dosages, diet, alcohol, and tobacco. However, it is believed that genetic differences also play an important role, both in the metabolization of the administered product (pharmacokinetics) and on its sensitivity (pharmacodynamics).

The study of genes involved in specific metabolic pathways and of the whole genome has a preventive purpose, with the aim of identifying early the patients’ response mechanisms to drugs and the relationships between human leukocyte antigens and any adverse reactions [31]. For example, patients carrying the HLA-B5701 variant (HLAB is the gene responsible for resistance to the HIV virus) more frequently experience unwanted reactions towards Abacavir. A total of 98% of patients with carbamazepine adverse reactions (Stevens-Johnson syndrome, Lyell syndrome, or toxic epidermal necrolysis) have HLA-B1502-mediated immunity (typically Asian patients) [29]. The same molecule is present only in 4.2% of subjects who do not have adverse reactions. The most accredited hypothesis is that the HLA-B1502 molecule is involved in a cell-mediated response by presenting, due to a binding affinity, toxic epitopes of the drug with subsequent activation of a cytolytic cascade [29].

Additionally, during the COVID-19 pandemic, an attempt was made to study the HLA allelic sequences in individuals who had contracted the disease [30]. Regarding type 2 diabetes, the response to drugs that stimulate insulin secretion by pancreatic cells or to increase insulin sensitivity is influenced by the polymorphism of the IRS1 Gly972Arg receptor (widespread in some populations, e.g., Italian and French) which contributes to the failure of some specific therapies by altering the link between the IRS1 substrate and other molecules that mediate the final effect of insulin [33].

Pharmacogenetic studies aim to identify the ideal dosages for each individual patient, to maximize the response, to optimize efficacy and safety, and to reduce unwanted side effects and damage. Future studies should aim to identify and distinguish genetically non-responsive or, on the contrary, hyperresponsive patients, to the point of avoiding drug-induced effects, attributable to genetically determined individual responses [41].

Pharmacogenetics and pharmacogenomics are substantially still experimental disciplines. There is a need to adapt to the rules governing the experimentation, remembering, however, that there are no shared criteria relating to the setting up and conduct of the research and there is a need for clear guidelines [42].

On the one hand, there is a need for accurate, rapid, safe, and protected diagnostic gene tests and not all diagnostic tests are validated. On the other hand, the genotyping of a single patient (genetics drugs response profile) requires the acquisition of delicate genetic information, the collection of which has important ethical and regulatory implications for the processing of ultra-sensitive data. Only very few drugs approved by the FDA then contain “pharmacogenomic” information [43]. There is a need to know the real clinical, analytical, and molecular genetic validity of the tests that can be performed and of personnel with great ability to interpret the results, both of which are crucial elements on the applicability of pharmacogenetic tests. There are also doubts about some biomarkers, also in relation to the aforementioned ethical and privacy issues [44].

The goal of pharmacogenetics is to reconstruct a gene profile for everyone so as to identify the “polymorphic variants” involved in the mechanism of action of drugs at the DNA level [42,43].

Therefore, there are problems not only regarding the acquisition, treatment, and storage of samples, but also the validity of the consent given by patients. The problem of the collection of ultra-sensitive data and the possible advantage for the single individual, goes hand in hand with that of the information to be provided, the public utility of the study and confidentiality, the resources used for the experimentation, the evaluation of the protocols, the evaluation and use of the results, the supervision and favorable opinion of the Ethics Committees, the possible presence of Sponsors, the publication of the results, and the responsibilities of the investigators [42]. The intervention of the Ethics Committees is required for any collection of human biological samples used for genetic testing, but the same Committees underline the need for reference guidelines and shared protocols regarding the conduct to be adopted.

It would be advisable to be able to standardize, through international guidelines, the methods of data collection and use of tests. Managing the collection and storage of data is difficult and not without problems. The analysis of an individual’s gene pool has strong ethical implications [44], whether the data is anonymous or whether it is identified and identifiable. Confidentiality linked to anonymization implies the impossibility of second thoughts and withdrawal of the sharing of intentions (consent) or, sometimes, of specific practical uses of the results, but guarantees acceptable privacy.

The information provided must indicate in detail the analysis to be carried out, the methods of storage of biological samples, communication of the test results, and the cost/benefit ratio [45,46]. Another aspect relates to the possibility of involuntary or casual finding of information about other collateral or coexisting pathologies, not directly related to the research. Then, questions also arise regarding the applicability of the results obtained and the regulation of this important research phase as well.

The Italian Society of Hospital Pharmacy, with the patronage of some scientific societies, has actively dealt with these problems. The EMA has also regulated the privacy issue of studies.

Surely, pharmacogenetic tests will allow personalized therapies in the future and will revolutionize the prescribing methods of drugs, but for now, genotyping is still experimental. The use of experimental branches of medicine and pharmacology is strictly regulated from a regulatory point of view, especially considering the sensitivity of the subject and the biomarkers used. A targeted decoding of the human genome could lead to great practical results in terms of population health, prescriptive appropriateness, but also a reduction in social costs and hospitalizations.

### 4.3. Boundary between Clinical Practice, Off-Label and Experimental Use

The use, prescription, and administration of drugs must consider the regulatory system and the specific AIFA authorization relating to therapeutic indications, methods, and timing of administration, shown in the technical data sheet of each product. It is not recommended to use drugs or medicinal substances with a different purpose, dosage or on a population other than the authorization of the Ministry of Health. Clinical trial projects must be adequately authorized and controlled according to the regulations in force on the subject (Ministry of Health Decree of 15 July 1997) and with the positive opinion of the relevant Bioethics Committees. These trials are governed by a series of very strict rules that safeguard and protect those who voluntarily accept to undergo a specific experimental program.

There is the so-called off-label use, that is the use of substances with different purposes, dosage, or population than those recognized. In essence, this is a use different from the authorizations of the Ministry of Health or different from that provided for in the list prepared by the Single Medicines Commission and by the national procedures of Authorization for Marketing, exceeding the scheme dosage and contraindications contemplated in the Summary of Product Characteristics [17], not systematic (otherwise it would fall under the legislation relating to clinical trials), but occasional and targeted and supported by clear information, guidelines, and evidence of efficacy and usefulness. The use of these products cannot be separated from the existence of specific evidence and from a more timely, precise, and correct information provided to the patient, prior to sharing the proposed therapeutic choice, and the lack of equally effective alternatives.

Obviously, the prescription and use of drugs in the off-label mode amplifies the risks of civil and penal liability, and the obligations of the healthcare professional in terms of anamnestic evaluation, adequacy of diagnosis, prescription/administration, and supervision.

The Penal Court of Cassation, with sentence no. 37077 of 30 September 2008 dealt with the issue of the penal relevance of the use of off-label drugs, noting that “any injuries resulting from administration can be attributed as fault to the responsibility of the doctor who ordered it, if the latter, even though the medical prescription was lawful, did not subject the effects of the treatment to scrupulous verification, in order to avoid exceeding the risk not permitted by the law, and did not comply with the limit determined by the risk/benefit ratio in using the drug”. In summary, the healthcare professional must not act “on an experimental basis” and must carry out a careful comparative analysis of the benefits and risks associated with the particular use of the drug, predictable based on the clinical situation of the specific patient. In the dental field, the classic example is metronidazole used in periodontology in case of infections caused by anaerobic bacteria that cannot otherwise be controlled.

## 5. Conclusions

Keeping up to date on recent medical and scientific acquisitions is a regulatory and ethical duty of healthcare professionals. Evidence tests associated with an expert, and prudent and diligent conduct of clinical activity in relation to the specific clinical case reduce the possibility of unwanted and adverse events, including in cases related to prescription/drug administration. The greater the knowledge of drugs, medical devices, and legal regulations, the fewer are the problems resulting from the burden of responding to patient’s non-compliance and the associated consequences. At the same time, the possibilities of therapeutic efficacy are greater.

The adequacy of pharmacological prescriptions is associated with a meticulous assessment of the patient’s general and specific health concerns, and with a deep and up-to-date knowledge of the aids, including pharmacological disposition of the healthcare professional. The contextual reduction of undesirable events produces a reduction in medico–legal problems and relapses in terms of patient dissatisfaction and litigation. The greater the knowledge of drugs and the regulatory and clinical provisions governing their use, the greater their effectiveness.

## Figures and Tables

**Table 1:** Some drugs commonly prescribed in dentistry practice related to side effects (see the *Data Availability Statement* section for more details).

Pathologies/Signs/Symptoms	Drug	Side Effects
Aphthae, desquamative gingivitis, mild multiforme erythema, lichen	Fluocinonide (Corticosteroid)	Dry or cracked mucosae; prolonged use awareness; edema
Lichen planus Lupus erythematosus Relapsing affections	Cyclosporine	Impaired renal function, hypertension, gingival enlargement
	Prednisone	Cardiac arrhythmias, adrenal insufficiency, development of cushingoid state, oropharyngeal candidiasis, pathologic fracture of long bones, anemia, lupus erythematosus-like lesions
Lupus erythematosus moderate to severe cases	Azathioprine (immunosuppressant, myelosuppressive)	Fever, bruising, bleeding, cytotoxicity
Herpes simplex Herpes zoster	Aciclovir (guanosine acid)	Nausea, vomiting, abdominal pain, diarrhea, fatigue, rash
Candida, prosthetic candidiasis, chronic plaque candidiasis, angular cheilitis, rhombic glossitis	Miconazole	Reduction in contraceptive efficacy
Pain/ Inflammation	Non Steroidal Anti-Inflammatory Drugs	Excessive bleeding in a patient taking oral anticoagulants, hypoglycemic crisis, excessive sleepiness (patients taking oral anticonvulsivants)

**Table 2:** Reports on the EudraVigilance database for some drugs commonly used in dentistry (see the *Data Availability Statement* section for more details).

Drug	Action	Reports
Tramadol	Narcotic analgesic	31,455
Norepinephrine	Vasoconstrictor/sympathomimetic	1594
Epinephrine	Vasoconstrictor/sympathomimetic	5277
Amoxicillin + clavulanate	Antibiotic (penicillin with β-lactamase inhibitor)	51,019
Metronidazole	Antibiotic (imidazole derivative)	15,332
Clarithromycin	Antibiotic (macrolide)	20,868
Doxycycline	Antibiotic (tetracycline)	10,373
Ketoprofen	NSAIDs	13,148
Ibuprofen	NSAIDs	48,904
Triamcinolone	Corticosteroid	5466
Bromazepam	Benzodiazepine	6976
Diazepam	Benzodiazepine	18,966
Denosumab	Anti-bone resorption monoclonal antibody	44,322

## Data Availability

Data available in a publicly accessible repository that does not issue DOIs. Publicly available datasets were analyzed in this study: Data reported in Table 1 were obtained by consulting the Drugs.com database for drug side effects. Drugs.com is an online pharmaceutical encyclopedia that provides drug information for consumers and healthcare professionals, primarily in the United States. Data sources include IBM Watson Micromedex (updated 1 November 2022), Cerner Multum™ (updated 25 October 2022), ASHP (updated 12 October 2022) and others. The website, accessed and consulted on 2 November 2022, can be found here: https://www.drugs.com/sfx/. Data reported in Table 2 were obtained using the online search engine of the EudraVigilance database (updated on 14 May 2022 and accessed on 17 May 2022), that can be found here: https://www.adrreports.eu/en/search.html (English lang.).
